# Safety and activity of the first-in-class locked nucleic acid (LNA) miR-221 selective inhibitor in refractory advanced cancer patients: a first-in-human, phase 1, open-label, dose-escalation study

**DOI:** 10.1186/s13045-023-01468-8

**Published:** 2023-06-26

**Authors:** Pierfrancesco Tassone, Maria Teresa Di Martino, Mariamena Arbitrio, Lucia Fiorillo, Nicoletta Staropoli, Domenico Ciliberto, Alessia Cordua, Francesca Scionti, Bernardo Bertucci, Angela Salvino, Mariangela Lopreiato, Fredrik Thunarf, Onofrio Cuomo, Maria Cristina Zito, Maria Rosanna De Fina, Amelia Brescia, Simona Gualtieri, Caterina Riillo, Francesco Manti, Daniele Caracciolo, Vito Barbieri, Eugenio Donato Di Paola, Adele Emanuela Di Francesco, Pierosandro Tagliaferri

**Affiliations:** 1 Department of Experimental and Clinical Medicine (DMSC), Magna Graecia University, Catanzaro, Italy; 2 Phase 1 and Translational Medical Oncology Unit, AOU Renato Dulbecco, Catanzaro, Italy; 3 Medical Oncology Unit, AOU Renato Dulbecco, Catanzaro, Italy; 4 Institute of Research and Biomedical Innovation (IRIB), Italian National Council (CNR), Catanzaro, Italy; 5 Radiology Unit, AOU Renato Dulbecco, Catanzaro, Italy; 6 Biometrics Department, LINK Medical Research AB, Uppsala, Sweden; 7 Pharmacy Unit, AOU Renato Dulbecco, Catanzaro, Italy; 8 Pharmacology Unit, Department of Science of Health, Magna Graecia University, Catanzaro, Italy

**Keywords:** miRNA therapeutics, microRNA, miRNA, RNA therapeutics, Non-coding RNA therapeutics, miR-221, LNA-i-miR-221, First-in-human, First-in-class, Phase 1, Cancer, Refractory, Advanced, Clinical trial

## Abstract

**Background:**

We developed a 13-mer locked nucleic acid (LNA) inhibitor of miR-221 (LNA-i-miR-221) with a full phosphorothioate (PS)-modified backbone. This agent downregulated miR-221, demonstrated anti-tumor activity against human xenografts in mice, and favorable toxicokinetics in rats and monkeys. Allometric interspecies scaling allowed us to define the first-in-class LNA-i-miR-221 safe starting dose for the clinical translation.

**Methods:**

In this first-in-human, open-label, dose-escalation phase 1 trial, we enrolled progressive cancer patients (aged ≥ 18 years) with ECOG 0–2 into 5 cohorts. The treatment cycle was based on a 30-min IV infusion of LNA-i-miR-221 on 4 consecutive days. Three patients within the first cohort were treated with 2 cycles (8 infusions), while 14 patients were treated with a single course (4 infusions); all patients were evaluated for phase 1 primary endpoint. The study was approved by the Ethics Committee and Regulatory Authorities (EudraCT 2017-002615-33).

**Results:**

Seventeen patients received the investigational treatment, and 16 were evaluable for response. LNA-i-miR-221 was well tolerated, with no grade 3–4 toxicity, and the MTD was not reached. We recorded stable disease (SD) in 8 (50.0%) patients and partial response (PR) in 1 (6.3%) colorectal cancer case (total SD + PR: 56.3%). Pharmacokinetics indicated non-linear drug concentration increase across the dose range. Pharmacodynamics demonstrated concentration-dependent downregulation of miR-221 and upregulation of its CDKN1B/p27 and PTEN canonical targets. Five mg/kg was defined as the recommended phase II dose.

**Conclusions:**

The excellent safety profile, the promising bio-modulator, and the anti-tumor activity offer the rationale for further clinical investigation of LNA-i-miR-221 (ClinTrials.Gov: NCT04811898).

**Supplementary Information:**

The online version contains supplementary material available at 10.1186/s13045-023-01468-8.

## Background

Non-coding RNAs (ncRNAs) have emerged as key regulators of crucial biological pathways driving cancer onset and progression. As such, ncRNAs have garnered major interest as targets for innovative therapeutic approaches to treat a range of human malignancies.

Of the different classes of ncRNAs, microRNAs (miRNAs) are the most well studied [1]. MiRNAs are a phylogenetically conserved subclass of short transcripts, 18–24 nucleotides in length, that can target hundreds of individual messenger RNAs (mRNAs) by partial base pairing to their 3ʹ untranslated region. In this way, miRNAs can control gene and protein expression, and subsequently regulate biological pathways that can be disrupted in cancer [2]. Many studies have demonstrated that deregulated miRNAs promote cancer onset and progression, acting as oncogenic drivers which can produce tumor addiction [3, 4]. Strong evidence supporting the inhibition of these oncogenic miRNAs as a therapeutic strategy has been generated by several in vitro and in vivo preclinical investigations [5, 6]. However, no clinical data supporting this approach have been reported in cancer patients to date (see Additional file 1: Supplementary information).

Among deregulated miRNAs, miR-221 has been widely investigated for its oncogenic role and as a promising therapeutic target [7]. MiR-221 is transcribed together with its paralogue miR-222 as primary miRNA (pri-miR), before processing to precursor miRNA (pre-miR), and the two miRNAs share the same seed sequence. Several miR-221 targets relevant for tumor cell growth and survival have been reported to be consistently and significantly downregulated in many solid tumors and haematological malignancies, and there is substantial preclinical evidence that inhibition of miR-221 effectively upregulates tumor suppressor proteins and, consequently, induces anti-tumor activity in vitro and in vivo, indicating the potential clinical value of this strategy [8–10].

The targeting of oncogenic miRNAs has been widely investigated using antisense oligonucleotides (ASOs). ASOs act as inhibitors by forming a duplex with the guide strand of the target miRNA via Watson–Crick base pairing, therefore preventing interaction between the miRNA and its target mRNAs. This leads to restoration of downstream tumor suppressor protein expression and function. However, translation of these agents to the clinic has been hampered by inefficient and unspecific target binding, which results in low biological activity and off-target effects that lead to toxicity. To overcome these limitations several approaches for chemical modification of ASO backbones have been introduced [11, 12].

To this end, we have developed a 13-mer locked nucleic acid (LNA) inhibitor of miR-221 with a fully phosphorothioate (PS)-modified backbone. This miRNA-221 inhibitor, named LNA-i-miR-221, demonstrated efficient downregulation of miR-221, upregulation of canonical miR-221 targets, and anti-tumor activity in several preclinical tumor models [10]. Moreover, LNA-i-miR-221 demonstrated favorable toxicokinetic profiles in mice, rats, and monkeys with rapid and wide tissue distribution [13], without behavioural changes and/or organ-related toxicity, in GLP animal studies [8, 10, 13, 14]. Furthermore, we investigated the protein binding of this new agent in rats, monkeys, and humans by generating an ultrafiltration method [14]. The integration of these findings into multiple allometric interspecies scaling methods allowed to infere the LNA-i-miR-221 pharmacokinetics in humans, thereby defining the safe starting and escalation doses for the first human clinical trial [14].

On these bases, we aimed to evaluate this first-in-class miR-221 inhibitor in advanced cancer patients in a phase I dose-escalation study. The primary objective of this study was the safety of LNA-i-miR-221, the identification of the maximum tolerated dose (MTD), and of the recommended dose for phase II trials (RP2D). Exploratory analysis of pharmacokinetics, pharmacodynamics, and of anti-tumor activity was also planned.

## Methods

### Study oversight

We enrolled patients with advanced solid tumors in a first-in-human, phase I, open-label, dose-escalation study. Patients were recruited at one site, the Magna Graecia University/AOU Mater Domini Hospital (now AOU Renato Dulbecco), Catanzaro, Italy. The study was approved by the Regulatory Authorities AIFA/ISS (Italy) in March 2018 and the local Ethics Committee in July 2018. The first patient was enrolled on February 2019, and the study was closed on December 2021. The study was registered during the trial conduction, after accrual of 10 patients (ClinTrials.Gov NCT04811898). The design of the trial is shown in the Additional file 1: Fig. S1. Additional information on study protocol is available online (access provided in the Additional file 1: Supplementary material). The study was conducted in accordance with the principles of the Declaration of Helsinki, Good Clinical Practice guidelines of the International Council for Harmonisation, and applicable laws. The study was fully academic, and funds were made available by “A Special Program Molecular Clinical Oncology—“5 × 1000” no. 9980 (2010-15) and its Extension Program no. 9980 (2016-17). Data were collected by the investigators, CRF stored according to GCP criteria, analysed by statisticians paid by the Institution, and interpreted by all the authors.

### Patients

The key eligibility criteria were male and female patients > 18 years old with histologically diagnosed malignancies, advanced and guideline treatment-resistant, with progressive disease. Laboratory and clinical data showing well-preserved organ functions were required, without signs of major general deterioration (ECOG 0–2 was eligible). All patients provided written informed consent.

In total, 24 patients were screened, and 19 patients were treated. Of these patients, 17 were found to be evaluable on the primary endpoint. Two patients were excluded from the analysis for limited data collection due to no completion of the scheduled 4 days treatment. Two additional patients were enrolled in the pre-planned expansion cohort. The median follow-up of treated patients was 56 days.

### Study treatment

Patients were enrolled in five dose-escalating cohorts. The initial study protocol included daily intravenous (IV) infusions on day 1 to 4 followed by a 24-day wash-out, before a second treatment cycle on day 29 to 32. Following completion of the first cohort, the study protocol was amended to reduce the treatment plan to a single treatment course and ensure patients adherence to protocol. Individuals included from cohort II to cohort V underwent 4 days of LNA-i-miR-221 administration on day 1 to 4, followed by 24 days observation.

LNA-i-miR-221 dose levels were determined using a modified Fibonacci 3 + 3 dose-escalation scheme with 3 patients enrolled in each cohort *plus* 3 additional patients in the case of one suffering grade 3–4 toxicity. The dose levels were (all in mg/kg body weight): cohort I: 0.5, cohort II: 1.0, cohort III: 2.0, cohort IV: 3.0, and cohort V: 5.0. An independent expansion cohort was also planned at the RP2D.

### Assessment and monitoring

The study began with a screening visit. Mandatory tumor assessment via computed tomography (CT) scan imaging was performed at enrolment and at cycle completion. Follow-up imaging was performed for evaluation of responses or clinical benefit. We selected to systematically perform CTscan for uniform clean data interpretation. CT assessment was performed on the 30th ± 1 day, as reported on V.E.S. (Visit Evaluation Schedule), except for patients belonging to the 1st cohort, whose CT evaluation was carried on 56th ± 1 day (the cohort has been completed before trial amendment). Patients returned to the clinic within 7 days from the baseline visit to receive the first infusion of LNA-i-miR-221 (day 1). Patients were admitted to the Phase I Center in-patients ward during days 1–4 due to the daily infusion of LNA-i-miR-221, the need of intensive monitoring, and the requirement of post-infusion blood and urine sampling.

Patients returned to the clinic at day 6, 8, 15, and 22 for clinical evaluation and blood collection. Final assessment and safety follow-up was performed on day 30 ± 1. At the same day, CT scan imaging was performed. Patients were scheduled for subsequent monthly visits. Imaging data were assessed according to the RECIST 1.1 criteria (version 2009) to determine anti-tumor activity. At each visit patient’s concomitant medications and adverse events (AEs) were registered.

### Pharmacokinetics (PK)

Blood sampling for PK was performed before beginning infusion and at 15 min, and 30 min post-beginning infusion, and at 30 min, 1 h, 2 h, 4 h, 6 h, 12 h, and 24 h post-infusion on days 1 and 2, and at 30 min post-beginning infusion, and at 1 h, 2 h, 4 h, and 24 h post-infusion on days 3 and 4, and then at 24 h (day 5) and 48 h (day 6) post the last infusion. Urine was collected before treatment and after 6, 12, and 24 h on day 1. Urine collection was then performed every 24 h until 48 h after the last infusion (day 6).

LNA-i-miR-221 concentrations were assessed by a previously developed and validated mass spectrometry analytical method [15] at Aptuit (Verona, Italy) and PK parameters were estimated using a non-compartmental approach (Phoenix WinNonlin software; version 6.4, Certara L.P). The analysis was performed from individual concentration–time profiles using the IV infusion model (200–202). Where applicable, the following PK parameters were reported: T_max_, C_max_, C_max_/Dose, T_last_, C_last_, λ_z_, t_½_, AUC_tlast_, AUC_tlast_/Dose, AUC_0-inf,_ Vz, Vss and Cl. Urine concentrations were measured within the same plasma sampling interval and used to assess LNA-i-miR-221 renal clearance.

### Pharmacodinamics (PD)

PD was performed by investigating target modulation in PBMCs. Expression levels of miR-221 and *CDKN1B* were determined by reverse transcription quantitative PCR (RT‐qPCR) in PBMCs isolated from patients at pre-dose (d1), and 24 h after LNA-miR-221 last infusion (d5). Data were normalized to U6 (miR-221) or *GAPDH* (*CDKN1B*). Results are expressed as 2^−△Ct^ or 2^−△△Ct^. Western blotting analysis of PTEN and p27 in representative protein samples extracted from PBMCs of patients before and after treatment was performed. β-actin was used as loading control.

### Study outcomes

The primary study endpoint was safety of LNA-i-miR-221, with assessment of the MTD, and of the RP2D. The MTD was defined as the dose below the dose level at which at least 2 out of 6 patients experienced dose-limiting toxicity (DLT).

The study exploratory endpoints were PK and PD profiles of LNA-i-miR-221, and preliminary investigation of anti-tumor activity, disease control and efficacy.

The NCI-Common Terminology Criteria for Adverse Events (CTCAE; version 4.03, 2010) was used to assess the severity of AEs (grade 1–grade 5). An AE was classified as DLT if the AE was graded 3 or 4 and assessed as having a potential relationship to LNA-i-miR-221 administration. An independent Safety Review Committee (SRC) reviewed all safety data on an ongoing basis. Dose-escalation to the next dose was permitted only after approval by the SRC.

### Statistical analysis

No formal sample size calculation was performed for this study. The sample size was based on historical norms for a study of this design and the 3 + 3 cohort scheme. All statistical analyses were performed using SAS® version 9.4 or higher (SAS Institute Inc., Cary, NC, USA).

## Results

### Patients

**Fig. 1:**
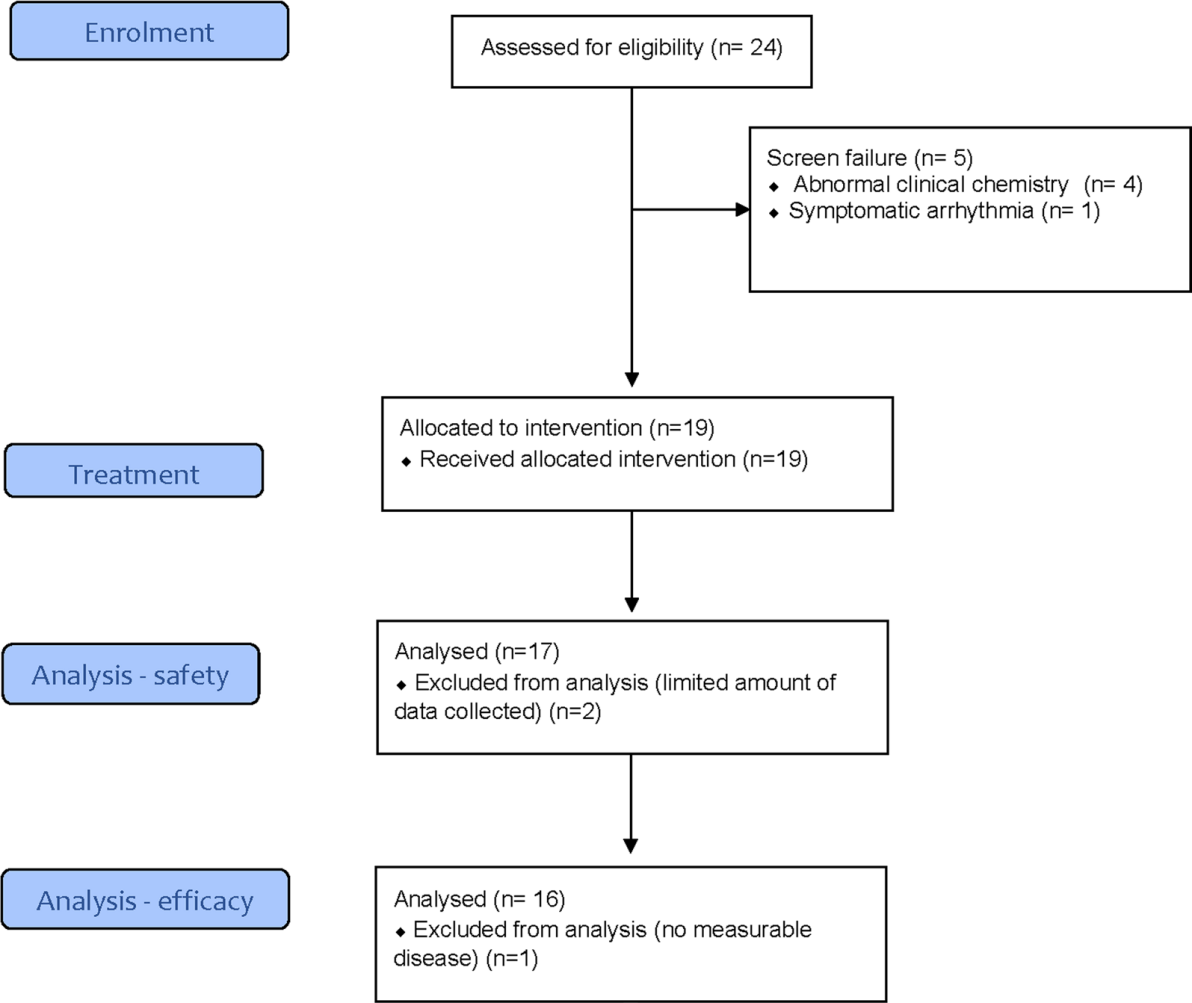
CONSORT diagram

Twenty-four patients with solid tumors were screened for eligibility and 19 of them underwent treatment. Screening failures were mainly due to abnormal clinical chemistry values (N = 4) and unstable symptomatic arrhythmia (N = 1). The first patient was enrolled on February 20, 2019 and the last patient’s last visit was on December 14, 2021. Two patients were excluded from the analysis. The safety data set included 17 patients. The activity analysis set included 16 patients, as one patient (the first enrolled), had no measurable disease. The CONSORT diagram is shown in Fig. 1. The complete study population and the patients baseline characteristics for each cohort are summarized in Table 1. Individual patient details are summarized in Additional file 1: Table S1 and S2.

**Table 1:** Baseline characteristics of the study population

Cohorts	0.5 mg/kg (N = 3)	1 mg/kg (N = 3)	2 mg/kg (N = 3)	3 mg/kg (N = 3)	5 mg/kg (N = 5)	Total (N = 17)
Age (years)						
Median	64	64	64	72	60	64
Q1, Q3	53, 72	62, 65	54, 67	39, 74	56, 62	56, 67
Sex						
Female	2 (66.7%)	3 (100%)	1 (33.3%)	2 (66.7%)	3 (60%)	11 (64.7%)
Male	1 (33.3%)	0	2 (66.7%)	1 (33.3%)	2 (40%)	6 (35.3%)
Body weight (kg)						
Median	70.0	61.0	67.0	58.2	68.2	68.0
Q1, Q3	60.5, 76.5	58.0, 62.0	63.5, 99.0	43.6, 72.0	68.2, 108.0	61.0, 72.0
Solid tumors occurring in the enrolled population						
Breast cancer	2 (66.7%)	0	1 (33.3%)	1 (33.3%)	1 (20%)	5 (29.4%)
Colorectal cancer	1 (33.3%)	2 (66.7%)	0	0	1 (20%)	4 (23.5%)
Gastric cancer	0	0	0	0	1 (20%)	1 (5.9%)
Glioblastoma	0	0	0	1 (33.3%)	0	1 (5.9%)
Hepatocellular carcinoma	0	0	0	0	1 (20%)	1 (5.9%)
Ovarian cancer	0	1 (33.3%)	0	0	0	1 (5.9%)
Pancreatic cancer	0	0	2 (66.7%)	0	1 (20%)	3 (17.6%)
Peritoneal mesothelioma	0	0	0	1 (33.3%)	0	1 (5.9%)
ECOG performance status						
0	3 (100%)	0	2 (66.7%)	0	5 (100%)	10 (58.8%)
1	0	3 (100%)	0	1 (33.3%)	0	4 (23.5%)
2	0	0	1 (33.3%)	2 (66.7%)	0	3 (17.6%)

Data are n (%), mean (SD; standard deviation). ECOG = Eastern Cooperative Oncology Group. N = number of subjects. Percentages are based on the number of subjects within each cohort

### Safety of LNA-i-miR-221

The primary endpoint of this study was the safety of LNA-i-miR-221. During the study no clinically significant changes in vital signs (heart rate and blood pressure) from baseline were noted. Additionally, no clinically significant changes in physical examination or ECG findings from baseline were reported during the study. Patients’ Performance Status ECOG scores during the study remained within the range 0–2. Furthermore, no clinically significant changes from baseline were noted in safety laboratory findings during the study.

Safety results are reported in Tables 2 and 3, providing an overview of the AEs. 37 AEs were reported by 15 patients. All AEs were CTCAE grade 0–2. The distribution of AEs in terms of System Organ Class, together with their CTCAE grade, is reported in Additional file 1: Tables S3 and S4. Gastrointestinal (m = 8) and nervous system (m = 8) were most frequent, followed by blood and lymphatic system AEs (m = 5).

**Table 2:** Overview of adverse events

Cohorts	0·5 mg/kg (N = 3)		1 mg/kg (N = 3)		2 mg/kg (N = 3)		3 mg/kg (N = 3)		5 mg/kg (N = 5)		Total (N = 17)	
	n (%)	M	n (%)	m	n (%)	m	n (%)	m	n (%)	m	n (%)	m
Any adverse event	2 (66·7%)	2	3 (100%)	7	2 (66·7%)	3	3 (100%)	16	5 (100%)	9	15 (88·2%)	37
Any serious adverse event	1 (33·3%)	1	0	0	0	0	1 (33·3%)	1	0	0	2 (11·8%)	2
Adverse events by severity												
Grade 0-1	0	0	3 (100%)	5	0	0	3 (100%)	8	5 (100%)	9	11 (64·7%)	22
Grade 2	2 (66·7%)	2	2 (66·7%)	2	2 (66·7%)	3	2 (66·7%)	8	0	0	8 (47·1%)	15
Grade > = 3	0	0	0	0	0	0	0	0	0	0	0	0
Adverse events by causality												
Suspected	0	0	0	0	0	0	0	0	0	0	0	0
Unsuspected	2 (66·7%)	2	3 (100%)	7	2 (66·7%)	3	3 (100%)	16	5 (100%)	9	15 (88·2%)	37

n = number of subjects, m = number of events

Percentages are based on the number of subjects within each cohort

**Table 3:** Listing of adverse events. Safety analysis set

Item	Grade 1–2 n (%)	Grade ≥ 3 n (%)
Any adverse event	15 (88.2%)	0
Vomiting	3 (17.6%)	0
Abdominal Pain	2 (11.8%)	0
Ascites	1 (5.9%)	0
Dyspepsia	1 (5.9%)	0
Nausea	1 (5.9%)	0
Paresthesia	2 (11.8%)	0
Headache	1 (5.9%)	0
Dizziness	1 (5.9%)	0
Seizure	1 (5.9%)	0
Anemia	4 (23.5%)	0
Platelet count decreased	1 (5.9%)	0
Fatigue	4 (23.5%)	0
Skin infection	2 (11.8%)	0
Agitation	1 (5.9%)	0
Confusion	1 (5.9%)	0
Pain of skin	1 (5.9%)	0
Pruritus	1 (5.9%)	0
Hypoxia	1 (5.9%)	0
Creatinine increased	1 (5.9%)	0
Hyperglicemia	1 (5.9%)	0
Neck pain	1 (5.9%)	0
Pain in extremity	1 (5.9%)	0
Tumor pain	1 (5.9%)	0

n = number of subjects

percetages are based on the number of subjects within each cohort

An additional primary aim of this study was to assess the MTD of LNA-i-miR-221 and the RP2D. To this end, patients were progressively enrolled into five dose-escalation cohorts. LNA-i-miR-221 was well tolerated at all dose levels, with no grade 3–4 dose-limiting toxicity (DLT) observed in any cohort.

### Pharmacokinetics (PK)

**Fig. 2:**
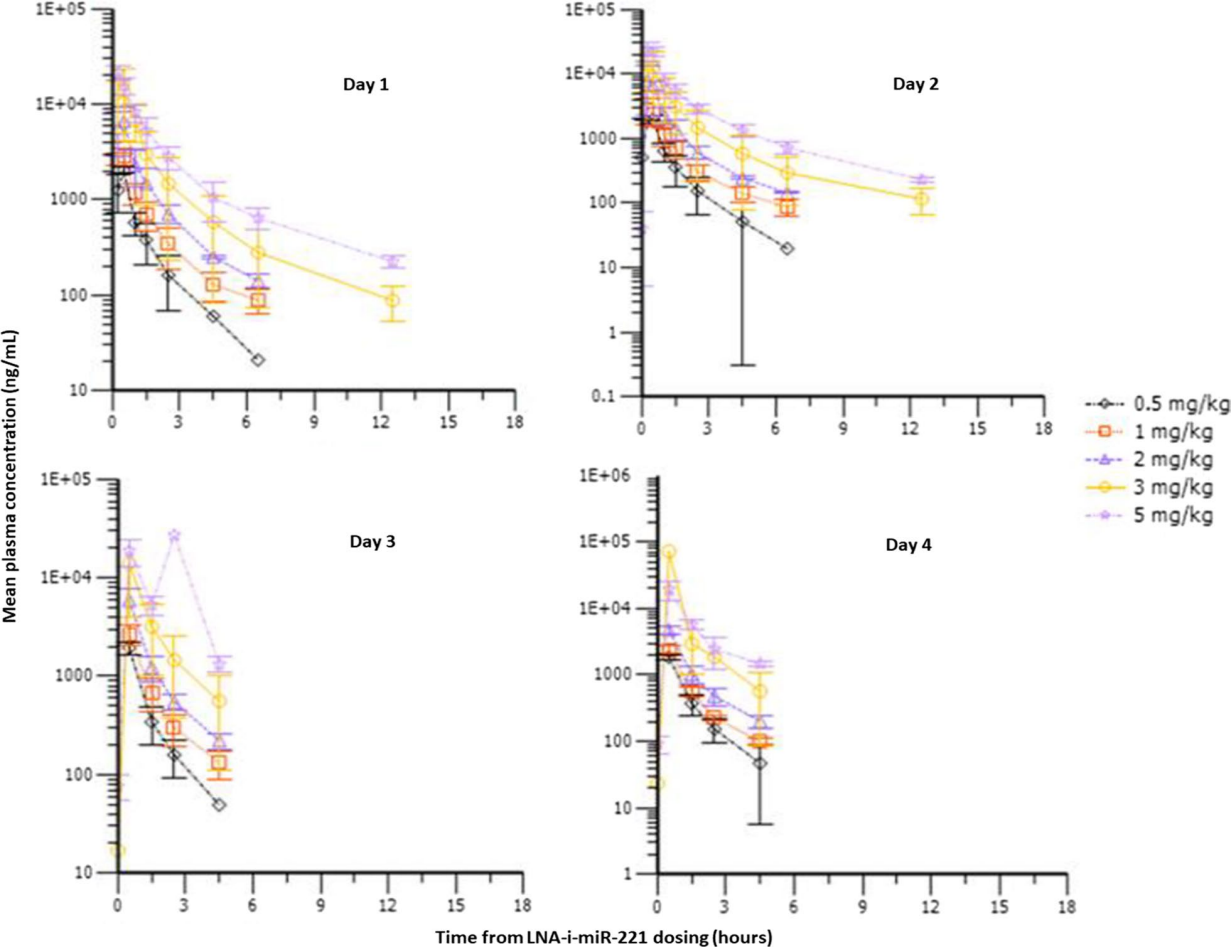
Serum concentration–time (geometric mean) profiles of LNA-i-mirR-221 by dose 0.5, 1, 2, 3 and 5 mg/kg, on day 1, 2, 3 and 4

Assessment of the PK profile of LNA-i-miR-221 was performed by mass spectrometry analysis of blood and urine samples collected on up to nine occasions up to 12 h post-infusion, using a previously developed analytical method [15]. The PK profile of LNA-i-miR-221 following administration of IV doses ranging from 0.5 mg/kg to 5 mg/kg are presented in Fig. 2. At all doses, LNA-i-miR-221 was rapidly cleared from the plasma compartment and distributed into tissues. As expected, peak plasma concentrations were observed immediately after dosing in most cases. On five separate instances T_max_ was seen later. This pattern was observed for the 3.0 mg/kg dose on day 3 and day 4, and the 5.0 mg/kg dose on days 2, 3 and 4. These observations are likely due to analytical variability as, in these cases, T_max_ was similar to the plasma concentration measured immediately following the end of the drug infusion.

Across all 4 LNA-i-miR-221 administrations in all cohorts, terminal plasma half-life harmonic mean values ranged from 1.1 to 4.9 h, increasing with the dose. This is unlikely to represent the true terminal half-life of LNA-i-miR-221 and more probably reflects the half-life of an initial distribution/elimination mixed phase. This interpretation is supported by the analysis of LNA-i-miR-221 levels in urine samples, where LNA-i-miR-221, dosed at 0.5 mg/kg, was detectable in the last urine samples collected after the day 4 dose (Additional file 1: Table S5). Additionally, analysis of LNA-i-miR-221 plasma concentrations indicate non-linear pharmacokinetics across the range of doses investigated in this study, based on the C_max_ and AUC_tlast_ values calculated for each dose. Peak C_max_ and AUC occurred with the 5 mg/kg dose, suggesting that, of the doses investigated, this dose may allow the most favorable tumor availability. Furthermore, comparison of C_max_ and AUC_tlast_ across consecutive doses suggests that no changes in systemic clearance patterns occurred. The summarized PK parameters, including C_max_, T_max_, AUC_tlast_, AUC_tlast/dose_, half-life, CI and Vz are presented in Additional file 1: Table S6.

### Pharmacodinamics (PD)

**Fig. 3:**
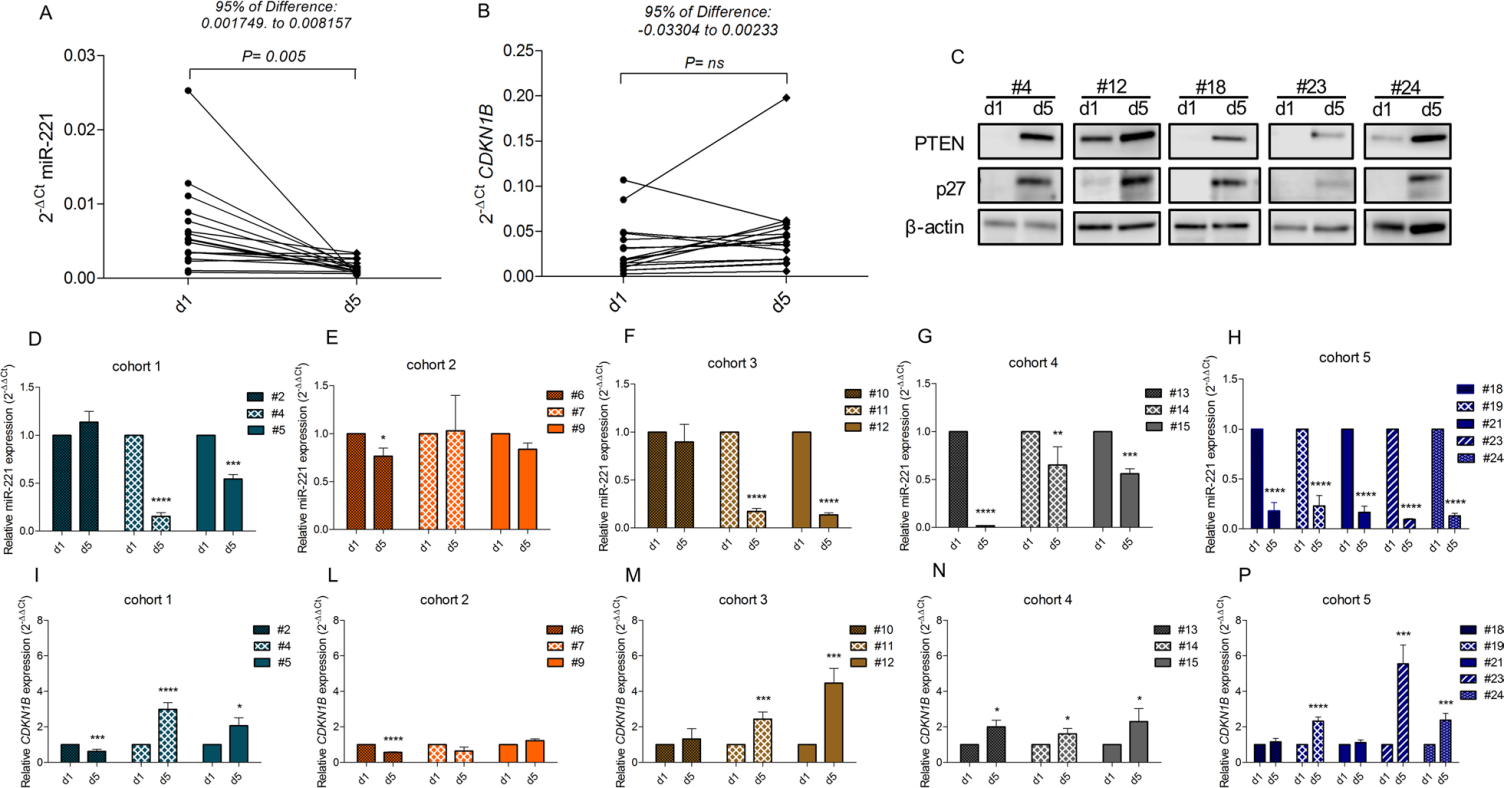
Target modulation on PBMCs by LNA-miR-221 inhibitor. Expression levels of miR-221 and *CDKN1B* were determined by reverse transcription quantitative PCR (RT‐qPCR) in PBMCs isolated from patients at pre-dose (d1) and 24 h after LNA-miR-221 end treatment (d5). Results are expressed as 2^−△Ct^ or 2^−△△Ct^. A and B show modulation of miR-221 and *CDKN1B* after LNA-miR-221 treatment (paired sample t test, P < 0.05; 95% CI: 95% confidence interval). In C, Western blotting analysis of PTEN and p27 in representative protein samples extracted from PBMCs of patients before and after treatment. D-P. Individual changes of expression level of miR-221 (D-H) and *CDKN1B* (I-P) in patients from each cohort treated with LNA-i-miR-221 regimen (unpaired sample t test, P: p-value; *P < 0.05, **P < 0.01, ***P < 0.001, ****P < 0.0001). Error bars represent mean ± standard deviation of each sample among triplicates

Assessment of the PD profile of LNA-i-miR-221 was performed by reverse transcription quantitative PCR (RT‐qPCR) of miR-221 and CDKN1B in PBMCs isolated from patients at pre-dose (d1) and 24 h after LNA-miR-221 end treatment (d5). Figure 3 shows strong downregulation of miR-221 during LNA-miR-221 treatment (paired sample t-test, P < 0.05; 95% CI: 95% confidence interval) (Fig. 3A, D–H). This effect was more pronounced with increased LNA-i-miR-221 doses reaching the maximum in the last and expansion cohort. In parallel, CDKN1B/p27 was upregulated at mRNA (Fig. 3B, I–P). Moreover, in some PBMC protein extract available samples from LNA-i-miR-221 treated patients, Western blotting analysis showed upregulation of PTEN and p27 as compared with pre-treatment controls (Fig. 3C). No changes in PBMC morphology, number and cell phenotype were observed.

### Anti-tumor activity of LNA-i-miR-221

Finally, exploratory analysis of the putative anti-tumor activity of LNA-i-miR-221 was also planned. The effect of administration of LNA-i-miR-221 on tumor size in each patient was determined by analysis of CT scan images, which were assessed in accordance with the RECIST 1.1 criteria. This analysis showed that eight patients had stable disease (SD) (50.0%) during the study, while seven patients (43.8%) had progressive disease (PD) (Table 4).

**Table 4:** RECIST 1.1. responses in patients evaluable for clinical activity

Response	0.5 mg/kg (N = 2)	1 mg/kg (N = 3)	2 mg/kg (N = 3)	3 mg/kg (N = 3)	5 mg/kg (N = 5)	Total (N = 16)
Response						
CR	0	0	0	0	0	0
PR	0	0	0	0	1 (20%)	1 (6.3%)
SD	1 (50.0%)	2 (66.7%)	2 (66.7%)	2 (66.7%)	1 (20%)	8 (50.0%)
PD	1 (50.0%)	1 (33.3%)	1 (33.3%)	1 (33.3%)	3 (60%)	7 (43.8%)

Percentages are based on the number of subjects within each cohort

CR = Complete response, PR = Partial response, SD = Stable disease, PD = Progressive disease

**Fig. 4:**
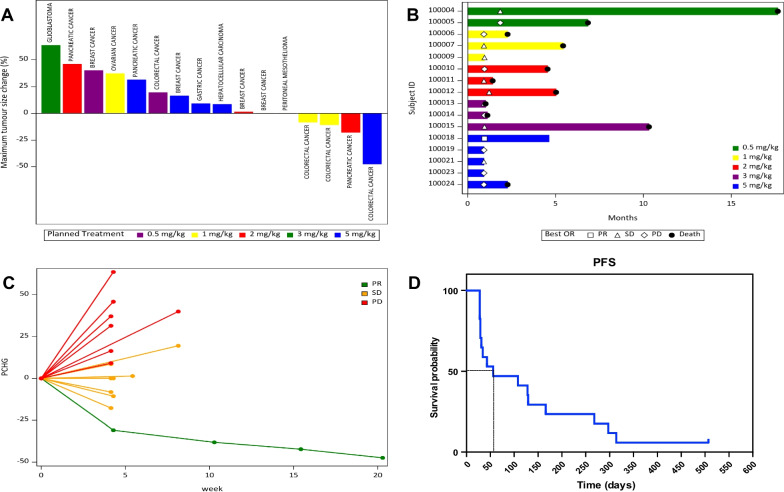
Tumor responses and patient outcomes during the course of this study: **A** Waterfall plot of CT scan findings according to modified Response Evaluation Criteria in Solid Tumors 1.1. **B** Swimmer plot of time on study treatment, response status, and survival. **C** Spider plot of tumor trajectories. **D** Kaplan–Meier (KM) curve of progression-free survival (PFS)

Except for the patient who received a 5 mg/kg dose and displayed a partial response (PR), there were no major differences in terms of tumor response among the cohorts. Figure 4A shows a waterfall plot of CT scan data for each cohort and tumor type, Fig. 4B shows a swimmer plot depicting time on study treatment, response status, and survival, Fig. 4C shows a spider plot of tumor trajectories colour-coded according to RECIST 1.1 and Fig. 4D shows the Kaplan–Meier (KM) curve of progression-free survival (PFS).

**Fig. 5:**
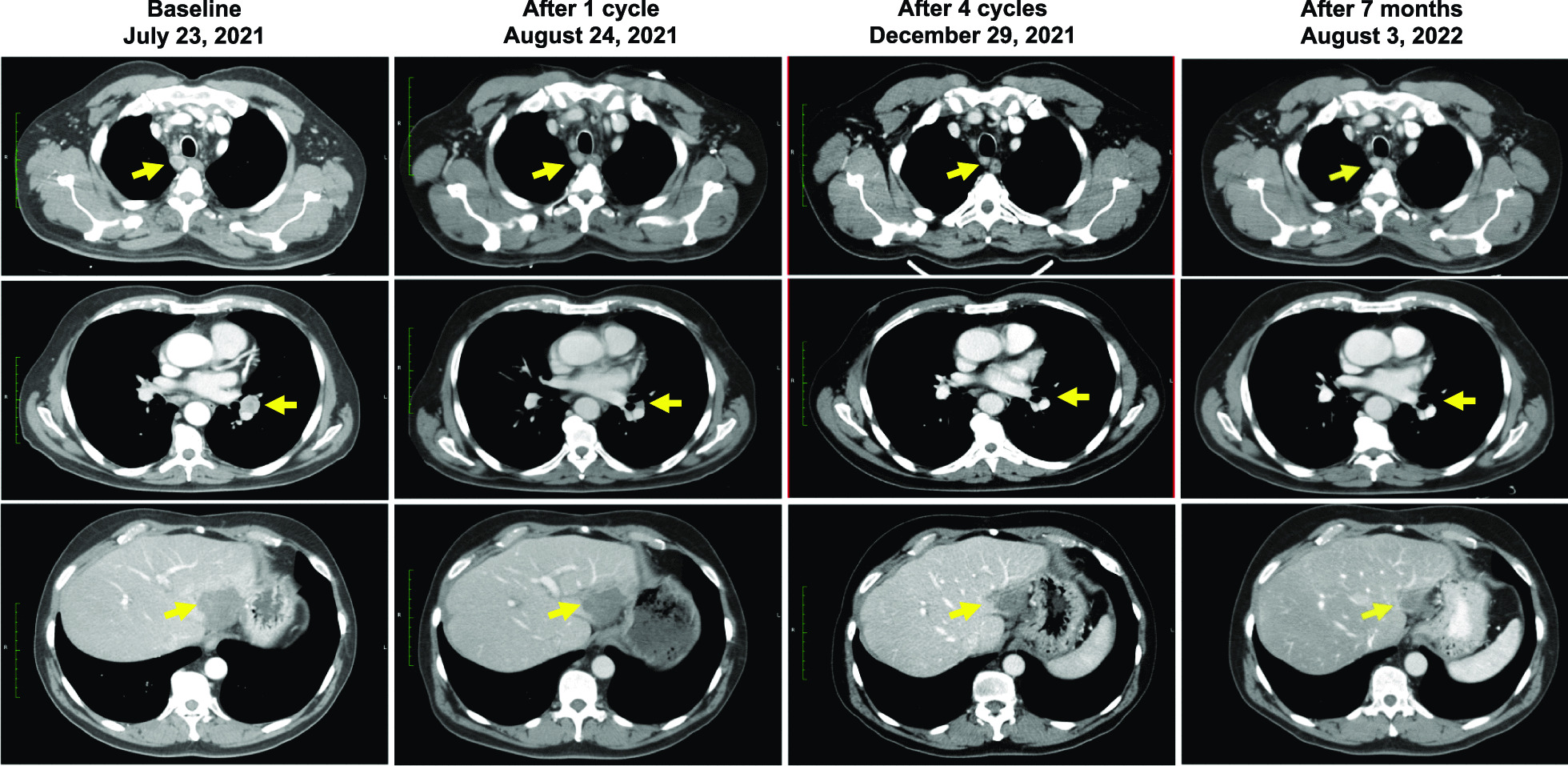
Progressive tumor shrinkage in a 60-year-old metastatic colon carcinoma patient with confirmed PR evaluated on three index lesions. The patient underwent left hemicolectomy in 2018 with histological evidence of moderately differentiated intestinal adenocarcinoma, RAS mutated. The patient was treated for stage IV disease with multiple regimens (FOLFOX plus Bevacizumab followed by surgical resection of liver metastases; FOLFIRI plus Aflibercept, FOLFOX, FOLFIRI rechallenges, Trifluridine/Tipiracil, and Regorafenib). The patient had disease progression and was enrolled within cohort V (5 mg/kg for four consecutive days). The RECIST 1.1 assessment after the first cycle with LNA-i-miR-221 showed a clinical benefit. The patient received three subsequent treatment cycles on a compassionate use basis (pre-planned in the study protocol for patients with clinical response). The CT-image shown in the Figure demonstrates disease regression at cycle 1 with deepening response after cycle 4. Further tumor regression of the residual liver lesion has been observed after 7 months of follow-up. At the present (May 2023) the patient is still alive with PS 0

The patient assessed with a PR was subsequently treated with three additional cycles of LNA-i-miR-221, on a compassionate use basis, at the same dose (5 mg/kg) as used in the escalation cohort. CT scan images showing tumor shrinkage over time in this patient are presented in Fig. 5.

We conclude that the MTD was not reached in this study and we set the RP2D at 5 mg/kg, considering the absence of major toxicity, the favourable PK profile, the more pronounced PD activity, and the occurrence of anti-tumor activity, as demonstrated by a persisting, and increasing major response.

## Discussion

RNA-based therapeutics hold promise as a broadly applicable and adaptable way of targeting almost any protein or genome region. These attributes are exemplified by the rapid design and development of mRNA-based COVID-19 vaccines [16, 17] and RNA-based therapies for a wide range of diseases and indications are currently under development [11]. RNA-based therapeutics that manipulate miRNA function can act either as steric blockers, preventing the interaction of a miRNA with its downstream mRNA target(s), or as mimics, restoring the function of a given miRNA [18–20].

As RNA-based molecules are negatively charged, which hampers uptake across cell membranes, and prone to degradation by a range of nucleases, a variety of chemical modifications have been proposed to enhance their PK and PD. Specifically, third-generation chemical modifications introduce changes to the furanose ring generating, for example, LNA ASOs. To date, the RNA therapeutics that have progressed to clinical testing make use of second- or third-generation chemical modifications. These investigational therapies include two anti-miR-122 inhibitors, RG-101 (an N-acetyl galactosamine-conjugated ASO) and miravirsen (SPC3649; a β-D-oxy-LNA), that have undergone clinical trials as novel therapeutics for hepatitis C virus (HCV) infection. In addition, anti-miR-92a (MRG-110) has been investigated as an anti-angiogenetic agent to improve wound healing (NCT03603431), and anti-miR-21 (RG-012) as a therapy for the prevention of kidney fibrosis in patients with Alport syndrome (NCT03373786). However, to our knowledge, no miRNA-targeting therapies have been investigated in cancer patients to date [21], as also demonstrated by a systematic review of literature (Additional file 1: Supplementary information).

In this first-in-human phase I study of LNA-i-miR-221, a novel 13-mer LNA inhibitor of miR-221, 4 consecutive daily-doses of 5 mg/kg, after dose escalation, was well tolerated. No clinically significant changes in vital signs, physical examination or ECG findings were observed, nor were any clinically significant changes from baseline noted in safety laboratory results. During the course of the study 37 AEs were reported by 15 patients, all of which were CTCAE grade 0–2, mostly without potential relationship to LNA-i-miR-221 administration. Therefore, no major safety concerns were noted in this trial, and we conclude that the MTD was not reached.

Investigation of the PK profile of LNA-i-miR-221 in plasma revealed that, on average, LNA-i-miR-221 exhibited non-linear PK across the range of doses explored. Similar observations were reported during preclinical safety studies conducted in rats [14, 22]. The terminal half-life of LNA-i-miR-221, based on plasma concentrations, was calculated to be 1.1–4.9 h, and increased with the dose administered. However, LNA-i-miR-221 was detectable in urine up to 2 days post-last dose in all patients in all cohorts. This suggests that the true half-life of LNA-i-miR-221 is longer than that determined from plasma concentrations. As no significant changes in systemic clearance were observed following repeated administrations, the excretion of LNA-i-miR-221 in urine, at late time points, seems to suggest systemic distribution and tissue retention of LNA-i-miR-221. This is consistent with the known serum and intracellular protein binding properties of PS-modified ASOs, as well as previously reported clearance kinetics for PS-modified ASOs [11, 12].

Importantly, systemic exposure to LNA-i-miR-221 led to downregulation of miR-221 expression levels as well as increased expression of CDKN1B at mRNA and protein levels, and of PTEN, demonstrating that the infused naked LNA-i-miR-221 was active in producing a PD target modulation in circulating cells. These data prove that the injected investigational drug is biologically active on the canonical miR-221 relevant targets and that this activity indeed correlates with a PK increase in the higher dose patient cohorts. We have not assessed target modulation in patient’s tumors. However, in vivo pre-clinical data in rodents demonstrated high and persistent bioavailability of LNA-i-miR-221 in solid tissues [13].

Of the 16 patients treated with LNA-i-miR-221 during this study who were evaluable for clinical response, the majority had SD (8 patients), as assessed through CT scan imaging of tumors over time. One 60-year-old metastatic colon carcinoma patient, treated with 5 mg/kg LNA-i-miR-221, exhibited a PR and received a further three cycles of treatment (4 cycles in total) on a compassionate use basis. Taken together, these findings suggest that LNA-i-miR-221 has anti-tumor activity. As of August 2022, 9 months after the last dose of LNA-i-miR-221, the patient who exhibited a PR was alive, with persisting symptomatic improvement and even with a more profound radiological response. At the end of May 2023, the patient is still alive with an excellent performance status (PS 0).

Notwithstanding the limitations of this small study population and limited cohort sizes used in this first-in-human study, the results presented here provide the basis for further clinical investigation of LNA-i-miR-221 for the treatment of solid tumors. This study provides preliminary evidence of anti-tumor activity in treatment-resistant advanced cancer patients, and, to our knowledge, this trial represents the first clinical evidence of miRNA inhibition as a safe and effective treatment for human malignancies.

## Conclusions

The promising results reported here warrant additional clinical studies. The favorable PK profile, the high bio-modulatory activity on canonical targets, the safety profile at 5 mg/kg dose of LNA-i-mirR-221, together with the clinical anti-tumor activity, indicate that this dose is appropriate for use in future clinical trials. Immediate development plans for LNA-i-miR-221 include the design and conduct of a phase II basket study to investigate the efficacy of LNA-i-miR-221 for the treatment of multiple solid tumor types in parallel. On this basis, this study acts as the first-in-human proof-of-concept study for the use of miRNA-targeting strategies in cancer and provides a framework for the future development of this first-in-class LNA inhibitor of miR-221.

## Supplementary Information

**Additional file 1. Supplementary information:** Systematic review of anti-miRNA therapeutics of human cancer. **Fig S1:** Dose-escalation phase I treatments according to a 3+3 design and Fibonacci dose-escalation protocol. **Table S1:** Patients, demography, pathology and stage. **Table S2:** Previous treatments. **Table S3:** AE distribution in terms of CTCAE System Organ Class. **Table S4:** Adverse events. Safety analysis set. **Table S5:** Analysis of LNA-i-miR-221 in urine, 0.5 mg/kg dose. A) Treatment Day 1, B) Treatment Day 4. **Table S6:** Mean plasma PK parameters for each LNA-i-miR-211 dose. Access Link to the Clinical Trial Protocol (Version 3.0).

## Data Availability

The datasets generated and/or analysed during the current study are not presently publicly available but will be available from the corresponding author on reasonable request.

## Abbreviations

LNA: Locked nucleic acid
PS: Phosphorothioate
aCP: Advanced cancer patients
SD: Stable disease
PR: Partial response
ncRNAs: Non-coding RNAs
pri-miR: Primary miRNA
pre-miR: Precursor miRNA
ASOs: Antisense oligonucleotides
GLP: Good laboratory practice
MTD: Maximum toreated dose
RP2D: Recommended dose for phase 2 trials
CT: Computed tomography
PK: Pharmacokinetics
PD: Pharmacodynamics
Tmax: Time to Maximum Plasma Concentration
Cmax: Maximum Plasma Concentration
Tlast: Time of Last Measurable Concentration
Clast: Last Measurable Concentration
λz: Individual estimate of the terminal elimination rate constant
t½: Terminal Elimination Half-Life
AUC: Area Under the Concentration–Time Curve
AUCtlast: AUC to the Last Measurable Concentration
AUC0-inf: AUC Extrapolated to Infinity
Vz: Volume of Distribution at the Terminal Elimination Phase
Vss: Volume of distribution at steady state
Cl: Clearance
AEs: Adverse events
DLT: Dose limiting toxicity
CTCAE: Common terminology criteria for adverse events
SRC: Safety review committee
