# Supplementary material for: Safety and activity of the first-in-class locked nucleic acid (LNA) miR-221 selective inhibitor in refractory advanced cancer patients: a first-in-human, phase 1, open-label, dose-escalation study
Source: J Hematol Oncol. 2023 Jun 26;16:68. doi: 10.1186/s13045-023-01468-8 (PMC10294514; doi:10.1186/s13045-023-01468-8)
Supplement: Supplementary file 1 — Additional file 1. Supplementary information: Systematic review of anti-miRNA therapeutics of human cancer. Fig S1: Dose-escalation phase I treatments according to a 3+3 design and Fibonacci dose-escalation protocol. Table S1: Patients, demography, pathology and stage. Table S2: Previous treatments. Table S3: AE distribution in terms of CTCAE System Organ Class. Table S4: Adverse events. Safety analysis set. Table S5: Analysis of LNA-i-miR-221 in urine, 0.5 mg/kg dose. A) Treatment Day 1, B) Treatment Day 4. Table S6: Mean plasma PK parameters for each LNA-i-miR-211 dose. Access Link to the Clinical Trial Protocol (Version 3.0). [file 13045_2023_1468_MOESM1_ESM.pdf]

Additional File 1
Supplementary Material
for

"Safety and activity of a first-in-class locked nucleic acid (LNA) miR-221 selective inhibitor in refractory advanced cancer patients: a first-in-human, phase 1, open-label, dose-escalation study"

Pierfrancesco Tassone[1,2], Maria Teresa Di Martino[1,2,3], Mariamena Arbitrio[4,2], Lucia Fiorillo[2,3], Nicoletta Staropoli[2,3], Domenico Ciliberto[2,3], Alessia Cordua[1], Francesca Scionti[1], Bernardo Bertucci[5], Angela Salvino[2,3], Mariangela Lopreiato[1], Fredrik Thunarf[6], Onofrio Cuomo[1], Maria Cristina Zito[7], Maria Rosanna De Fina[7], Amelia Brescia[7], Simona Gualtieri[2,3], Caterina Riillo[1], Francesco Manti[5], Daniele Caracciolo[1], Vito Barbieri[3], Eugenio Donato di Paola[8], Adele Emanuela De Francesco[7], Pierosandro Tagliaferri[1,3]

[1]Department of Experimental and Clinical Medicine (DMSC), Magna Graecia University, Catanzaro, Italy
[2]Phase 1 and Translational Medical Oncology Unit, AOU Renato Dulbecco, Catanzaro, Italy
[3]Medical Oncology Unit, AOU Renato Dulbecco, Catanzaro, Italy
[4]Institute of Research and Biomedical Innovation (IRIB), Italian National Council (CNR), Catanzaro, Italy
[5]Radiology Unit, AOU Renato Dulbecco, Catanzaro, Italy
[6]Biometrics Department, LINK Medical Research AB, Sweden
[7]Pharmacy Unit, AOU Renato Dulbecco, Catanzaro, Italy
[8]Pharmacology Unit, Department of Science of Health, Magna Graecia University, Catanzaro, Italy

Corresponding author:
Prof. Pierfrancesco Tassone
Department of Experimental and Clinical Medicine (DMSC)
Magna Graecia University, Catanzaro, Italy
Tel: + 39 0961 3647029
E-mail: tassone@unicz.it

Table of contents

Supplementary information: Systematic review of anti-miRNA therapeutics of human cancer. p.3

Supplementary Figure 1. Dose-escalation phase I treatments according to a 3+3 design and Fibonacci dose-escalation protocol p.4

Supplementary Table 1. Patients, demography, pathology and stage p.5

Supplementary Table 2. Previous treatments p.6

Supplementary Table 3. AE distribution in terms of CTCAE System Organ Class p.8

Supplementary Table 4. Adverse events. Safety analysis set p.9

Supplementary Table 5. Analysis of LNA-i-miR-221 in urine, 0.5 mg/kg dose. A) Treatment Day 1, B) Treatment Day 4 p.10

Supplementary Table 6. Mean plasma PK parameters for each LNA-i-miR-211 dose p.11

Access Link to the Clinical Trial Protocol (Version 3.0) p.12

Systematic review of anti-miRNA therapeutics of human cancer

There is substantial preclinical evidence of the efficacy of miR-221 inhibitors as anti-tumour agents, gained from *in vitro* and *in vivo* studies on a variety of malignancies. These data support the clinical investigation of miR-221-silencing strategies as a novel approach for the treatment of human cancers. We performed a systematic review of the literature, searching PubMed for articles published in English between January 1, 2012, and February 10 2022, using the terms “microRNA”, “miRNA”, “miR”, “miRNA therapeutics”, “miR-221”, “miR-221 inhibitor”, “phase 1”, “first-in-human”, “relapsed”, “refractory”, “RNA therapeutics”, “ASO” “antisense oligonucleotides”, “LNA”, “LNA-i-miR-221”, “locked nucleic acid”, “LNA (locked nucleic acid)”, “dose finding”, and “ASO (antisense oligonucleotides)”. This search retrieved no previous reports of early (Phase I) or Phase II or III clinical studies on miR-221 inhibitors in cancer patients. Indeed, we found no previous clinical studies on the use of miRNA inhibitors in cancer treatment.

Supplementary Figure 1. Dose-escalation phase I treatments according to a 3+3 design and Fibonacci dose-escalation protocol

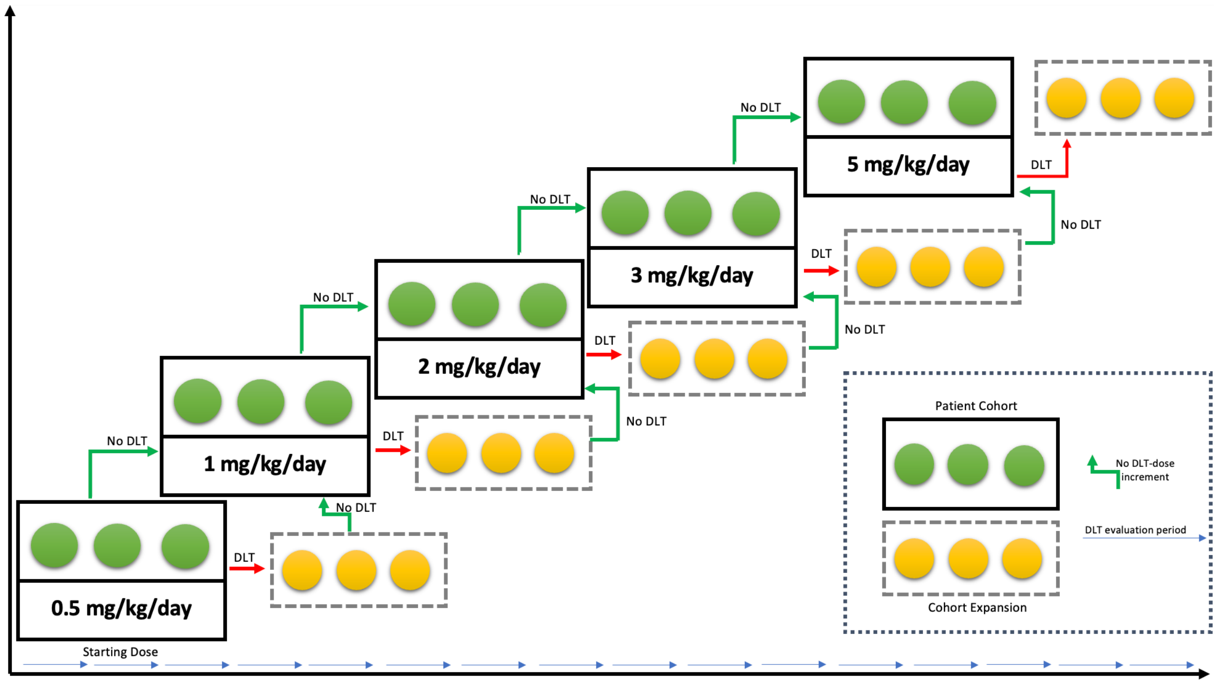

Supplementary Table 1. Patients, demography, pathology and stage

Cohorts	ID	Sex	Age (y)	Solid Tumors	Histology	Stage	Disease sites
**1**	**02**	F	72	Breast cancer	Infiltrating lobular adenocarcinoma (G3)	IV	Lymph node, Pleura
	**04**	M	65	Colorectal cancer	Adenocarcinoma (G2)	IV	Liver, Lung, Lymph node
	**05**	F	53	Breast cancer	Infiltrating adenocarcinoma (G3)	IV	Lung, Bone, Brain
**2**	**06**	F	62	Ovarian cancer	High grade serous carcinoma	IV	Liver, Lung, Lymph node, Pleura, Peritoneum
	**07**	F	64	Colorectal cancer	Adenocarcinoma	IV	Liver, Lung
	**09**	F	65	Colorectal cancer	Intestinal adenocarcinoma (G3)	IV	Lung, Lymph node
**3**	**10**	M	67	Pancreatic cancer	Adenocarcinoma	IV	Liver, Spleen, Adrenal gland, Brain
	**11**	M	64	Pancreatic cancer	Adenocarcinoma	IV	Liver, Peritoneum
	**12**	M	54	Breast cancer	Infiltrating ductal adenocarcinoma (G3)	IV	Lung, Pleura
**4**	**13**	F	39	Breast cancer	Infiltrating ductal adenocarcinoma (G3)	IV	Liver, Cerebellum, Spine
	**14**	M	72	Glioblastoma	Glioblastoma	Locally Advanced	
	**15**	F	74	Peritoneal mesothelioma	Peritoneal malignant mesothelioma	Locally Advanced	
**5**	**18**	M	60	Colorectal cancer	Adenocarcinoma	IV	Liver, Lymph node
	**19**	F	56	Pancreatic cancer	Adenocarcinoma	IV	Liver, Lymph node
	**21**	F	68	Hepatocellular carcinoma	Hepatocellular carcinoma (G2-3)	IV	Lung
	**23**	M	62	Gastric cancer	Adenocarcinoma	IV	Abdominal wall
	**24**	F	47	Breast cancer	Infiltrating ductal adenocarcinoma (G2)	IV	Liver, Lymph node, Breast, Brain

Supplementary Table 2. Patients' previous treatments

Patient ID	Treatments
**Patient ID #02**	- CMF (Cyclophosphamide plus Methotrexate plus Fluorouracil)<br>- FEC (Fluorouracil plus Epirubicin plus Cyclophosphamide)<br>- Tamoxifen<br>- Docetaxel<br>- Letrozole<br>- Pegylated Liposomal Doxorubicin<br>- Gemcitabine plus Vinorelbine<br>- Paclitaxel<br>- Capecitabine<br>- Capecitabine plus Cyclophosphamide<br>- Nab-paclitaxel<br>- Fulvestrant plus Palbociclib
**Patient ID #04**	- FOLFOX (Fluorouracil, Oxaliplatin, Calcium Levofolinate) plus Panitumumab<br>- Fluorouracil plus Panitumumab<br>- FOLFIRI (Fluorouracil, Irinotecan, Calcium Levofolinate) plus Bevacizumab<br>- Fluorouracil plus Bevacizumab<br>- Regorafenib<br>- Trifluridine/Tipiracil
**Patient ID #05**	- Doxorubicin plus Cyclophosphamide<br>- Paclitaxel<br>- Anastrozole<br>- Capecitabine<br>- Gemcitabine plus Vinorelbine<br>- Carboplatin plus Paclitaxel<br>- Eribulin
**Patient ID #06**	- Carboplatin plus Paclitaxel plus Bevacizumab<br>- Bevacizumab<br>- Carboplatin plus Liposomal Pegylated Doxorubicin<br>- Carboplatin plus Gemcitabine<br>- Carboplatin<br>- Niraparib<br>- Paclitaxel<br>- Topotecan<br>- Cyclophosphamide<br>- Gemcitabine<br>- Melphalan
**Patient ID #07**	- FOLFOX6 (Fluorouracil, Oxaliplatin, Calcium Levofolinate) plus Bevacizumab<br>- Capecitabine plus Bevacizumab<br>- Capecitabine<br>- Irinotecan<br>- FOLFOX (Fluorouracil, Oxaliplatin, Calcium Levofolinate)<br>- Regorafenib<br>- Trifluridine/Tipiracil
**Patient ID #09**	- Capecitabine plus Oxaliplatin<br>- Capecitabine<br>- FOLFIRI (Fluorouracil, Irinotecan, Calcium Levofolinate)<br>- Regorafenib
**Patient ID #10**	- Modified FOLFIRINOX (Fluorouracil, Oxaliplatin, Irinotecan, Calcium Levofolinate)<br>- Gemcitabine plus Nab-paclitaxel
**Patient ID #11**	- FOLFIRINOX (Fluorouracil, Oxaliplatin, Irinotecan, Calcium Levofolinate)<br>- Fluorouracil<br>- Gemcitabine plus Nab-paclitaxel
**Patient ID #12**	- TAC (Docetaxel, Doxorubicin and Cyclophosphamide)<br>- Tamoxifene plus Goserelin<br>- Docetaxel plus Trastuzumab plus Pertuzumab<br>- Capecitabine plus Lapatinib<br>- Letrozole plus Trastuzumab plus Triptoreline<br>- Trastuzumab emtansine<br>- Fulvestrant<br>- Margetuximab plus Vinorelbine

	- Paclitaxel plus Trastuzumab<br>- Palbociclib plus Fulvestrant<br>- Eribulin plus Trastuzumab<br>- Carboplatin plus Gemcitabine
**Patient ID #13**	- Paclitaxel plus Trastuzumab plus Pertuzumab<br>- Trastuzumab emtansine<br>- Capecitabine plus Lapatinib<br>- Trastuzumab plus Gemcitabine plus Vinorelbine<br>- Trastuzumab
**Patient ID #14**	- Temozolomide<br>- Regorafenib
**Patient ID #15**	- Carboplatin plus Pemetrexed<br>- Gemcitabine
**Patient ID #18**	- FOLFOX (Fluorouracil, Oxaliplatin, Calcium Levofolinate) plus Bevacizumab<br>- FOLFIRI (Fluorouracil, Irinotecan, Calcium Levofolinate) plus Aflibercept<br>- FOLFOX (Fluorouracil, Oxaliplatin, Calcium Levofolinate)<br>- FOLFIRI (Fluorouracil, Irinotecan, Calcium Levofolinate)<br>- Trifluridine/Tipiracil<br>- Regorafenib
**Patient ID #19**	- FOLFIRINOX (Fluorouracil, Oxaliplatin, Irinotecan, Calcium Levofolinate)<br>- FOLFIRI (Fluorouracil, Irinotecan, Calcium Levofolinate)<br>- FOLFOX (Fluorouracil, Oxaliplatin, Calcium Levofolinate)
**Patient ID #21**	- Sorafenib<br>- Regorafenib<br>- Cabozantinib
**Patient ID #23**	- FLOT (Fluorouracil, Oxaliplatin, Calcium Levofolinate, Docetaxel)<br>- FOLFOX (Fluorouracil, Oxaliplatin, Calcium Levofolinate)<br>- FOLFIRI (Fluorouracil, Irinotecan, Calcium Levofolinate)<br>- Paclitaxel plus Ramucirumab
**Patient ID #24**	- Paclitaxel<br>- Palbociclib plus Letrozole plus Triptorelin<br>- Capivasertib or Placebo plus Fulvestrant (Clinical Trial)<br>- Doxorubicin plus Cyclophosphamide<br>- Gemcitabine plus Vinorelbine

Supplementary Table 3. AE distribution in terms of CTCAE System Organ Class

**System Organ Class *(preferred terms reported)***	**AEs reported (m=37)**	**CTCAE Grade**
Gastrointestinal disorders *(vomiting, abdominal pain, ascites, dyspepsia, nausea)*	8 (21·6%)	0-1, 2
Nervous system disorder *(paresthesia, lethargy, headache, dizziness, seizure)*	8 (21·6%)	0-1, 2
Blood and lymphatic system disorders *(anemia, platelet count decreased)*	5 (13·5%)	0-1, 2
General disorders and administration site conditions *(fatigue)*	3 (8·1%)	0-1, 2
Infections and infestations *(skin infection)*	2 (5·4%)	0-1, 2
Psychiatric disorders *(agitation, confusion)*	2 (5·4%)	0-1, 2
Skin and subcutaneous tissue disorder *(pain of skin, pruritus)*	2 (5·4%)	0-1
Musculoskeletal and connective tissue disorder *(neck pain, pain in extremity)*	2 (5·4%)	0-1
Respiratory, thoracic and mediastinal disorders *(hypoxia)*	2 (5·4%)	2
Investigations *(creatinine increased)*	1 (2·7%)	0-1
Metabolism and nutrition disorders *(hyperglycemia)*	1 (2·7%)	0-1
Neoplasms benign, malignant and unspecified *(tumour pain)*	1 (2·7%)	2
m = number of events<br>Percentages are based on the total number of AEs reported		

Supplementary Table 4. Adverse events. Safety analysis set

	0.5 mg/kg (N=3)		1 mg/kg (N=3)		2 mg/kg (N=3)		3 mg/kg (N=3)		5 mg/kg (N=5)		Total (N=17)	
System Organ Class/Preferred Term	n (%)	m	n (%)	m	n (%)	m	n (%)	m	n (%)	m	n (%)	m
**Any adverse event**	**2 (66.7%)**	**2**	**3 (100.0%)**	**7**	**2 (66.7%)**	**3**	**3 (100.0%)**	**16**	**5 (100.0%)**	**9**	**15 (88.2%)**	**37**
**Gastrointestinal disorders**	**0**	**0**	**1 (33.3%)**	**2**	**2 (66.7%)**	**2**	**2 (66.7%)**	**2**	**2 (40.0%)**	**2**	**7 (41.2%)**	**8**
Vomiting	0	0	1 (33.3%)	1	0	0	1 (33.3%)	1	1 (20.0%)	1	3 (17.6%)	3
Abdominal pain	0	0	0	0	1 (33.3%)	1	0	0	1 (20.0%)	1	2 (11.8%)	2
Ascites	0	0	0	0	1 (33.3%)	1	0	0	0	0	1 (5.9%)	1
Dyspepsia	0	0	0	0	0	0	1 (33.3%)	1	0	0	1 (5.9%)	1
Nausea	0	0	1 (33.3%)	1	0	0	0	0	0	0	1 (5.9%)	1
**Nervous system disorder**	**0**	**0**	**0**	**0**	**0**	**0**	**1 (33.3%)**	**4**	**3 (60.0%)**	**3**	**4 (23.5%)**	**7**
Paresthesia	0	0	0	0	0	0	0	0	2 (40.0%)	2	2 (11.8%)	2
Headache	0	0	0	0	0	0	1 (33.3%)	3	0	0	1 (5.9%)	3
Dizziness	0	0	0	0	0	0	0	0	1 (20.0%)	1	1 (5.9%)	1
Seizure	0	0	0	0	0	0	1 (33.3%)	1	0	0	1 (5.9%)	1
**Blood and lymphatic system disorders**	**1 (33.3%)**	**1**	**2 (66.7%)**	**3**	**1 (33.3%)**	**1**	**0**	**0**	**0**	**0**	**4 (23.5%)**	**5**
Anemia	1 (33.3%)	1	2 (66.7%)	2	1 (33.3%)	1	0	0	0	0	4 (23.5%)	4
Platelet count decreased	0	0	1 (33.3%)	1	0	0	0	0	0	0	1 (5.9%)	1
**General disorders and administration site conditions**	**0**	**0**	**1 (33.3%)**	**1**	**0**	**0**	**2 (66.7%)**	**2**	**1 (20.0%)**	**1**	**4 (23.5%)**	**4**
Fatigue	0	0	1 (33.3%)	1	0	0	2 (66.7%)	2	1 (20.0%)	1	4 (23.5%)	4
**Infections and infestations**	**1 (33.3%)**	**1**	**0**	**0**	**0**	**0**	**0**	**0**	**1 (20.0%)**	**1**	**2 (11.8%)**	**2**
Skin infection	1 (33.3%)	1	0	0	0	0	0	0	1 (20.0%)	1	2 (11.8%)	2
**Psychiatric disorders**	**0**	**0**	**0**	**0**	**0**	**0**	**2 (66.7%)**	**2**	**0**	**0**	**2 (11.8%)**	**2**
Agitation	0	0	0	0	0	0	1 (33.3%)	1	0	0	1 (5.9%)	1
Confusion	0	0	0	0	0	0	1 (33.3%)	1	0	0	1 (5.9%)	1
**Skin and subcutaneous tissue disorder**	**0**	**0**	**0**	**0**	**0**	**0**	**1 (33.3%)**	**1**	**1 (20.0%)**	**1**	**2 (11.8%)**	**2**
Pain of skin	0	0	0	0	0	0	0	0	1 (20.0%)	1	1 (5.9%)	1
Pruritus	0	0	0	0	0	0	1 (33.3%)	1	0	0	1 (5.9%)	1
**Respiratory, thoracic and mediastinal disorders**	**0**	**0**	**0**	**0**	**0**	**0**	**1 (33.3%)**	**2**	**0**	**0**	**1 (5.9%)**	**2**
Hypoxia	0	0	0	0	0	0	1 (33.3%)	2	0	0	1 (5.9%)	2
**Investigations**	**0**	**0**	**1 (33.3%)**	**1**	**0**	**0**	**0**	**0**	**0**	**0**	**1 (5.9%)**	**1**
Creatinine increased	0	0	1 (33.3%)	1	0	0	0	0	0	0	1 (5.9%)	1
**Metabolism and nutrition disorders**	**0**	**0**	**0**	**0**	**0**	**0**	**0**	**0**	**1 (20.0%)**	**1**	**1 (5.9%)**	**1**
Hypeglycemia	0	0	0	0	0	0	0	0	1 (20.0%)	1	1 (5.9%)	1
**Musculoskeletal and connective tissue disorder**	**0**	**0**	**0**	**0**	**0**	**0**	**1 (33.3%)**	**1**	**0**	**0**	**1 (5.9%)**	**1**
Neck pain	0	0	0	0	0	0	1 (33.3%)	1	0	0	1 (5.9%)	1
**Musculoskeltal and connective tissue disorder**	**0**	**0**	**0**	**0**	**0**	**0**	**1 (33.3%)**	**1**	**0**	**0**	**1 (5.9%)**	**1**
Pain in extremity	0	0	0	0	0	0	1 (33.3%)	1	0	0	1 (5.9%)	1
**Neoplasms benign, malignant and unspecified**	**0**	**0**	**0**	**0**	**0**	**0**	**1 (33.3%)**	**1**	**0**	**0**	**1 (5.9%)**	**1**
Tumor pain	0	0	0	0	0	0	1 (33.3%)	1	0	0	1 (5.9%)	1

Supplementary Table 5. Analysis of LNA-i-miR-221 in urine, 0.5 mg/kg dose.

A) Treatment Day 1, B) Treatment Day 4 BLQ, below limit of quantification, ··, not applicable or data missing.

A

Dose 0.5 mg/kg		Day 1		
		Time of collection		
		Predose	6 hours	12 hours
Occasion	Subject identifier	Urine concentration	Urine concentration	Urine concentration
		(ng/mL)	(ng*mL/h)	(ng*mL/h)
1	2	BLQ	17·8	14·4
	4	BLQ	19·4	··
	5	BLQ	22·6	··
	Mean	··	19·9	4·78
	SD	··	2·42	··
	CV%	··	12	··

B

Dose 0.5 mg/kg		Day 4	Day 5	Day 6
		Time of collection		
			24 hours	48 hours
Occasion	Subject identifier	Urine concentration	Urine concentration	Urine concentration
		(ng/mL)	(ng/mL)	(ng/mL)
4	2	689	572	903
	4	613	1300	663
	5	726	813	853
	Mean	676	894	806
	SD	57·7	369	127
	CV%	9	41	16

Supplementary Table 6. Mean plasma PK parameters for each LNA-i-miR-221 dose

Dose (mg/kg)	Cohort	T_{max}* (hours)	C_{max} (ng/mL)	HL Lambda z* (hours)	AUC_{last} (h*ng/ml)	AUC_{tlast}/Dose (mg/ml)/(mg/kg)	Cl (ml/h/kg)	Vz (ml/kg)
0·5	I	0·5	1966·6	1·1	1752	3451	274	366
1·0	II	0·5	2693·2	1·5	3004	3125	310	713
2·0	III	0·5	5774·7	1·3	6080	3040	309	663
3·0	IV	0·5	10840·3	2·9	12331	4110	231	869
5·0	V	0·5	20979·2	4·9	33034	6607	157	1227

*median

Access to the Clinical Trial Protocol document (version 3.0):

https://oncologia.unicz.it/wp-content/uploads/2022/05/Phase1_clinical_protocol_LNA-i-miR-221_V-3.0.pdf.
